# Canine Gastrointestinal Nematodiases and Associated Risk Factors in Kigali City, Rwanda

**DOI:** 10.1155/2021/9956256

**Published:** 2021-07-23

**Authors:** Pie Ntampaka, François Niragire, Philip Njeru Nyaga, Gervais Habarugira

**Affiliations:** ^1^Department of Veterinary Medicine, University of Rwanda, P.O. Box 57, Nyagatare, Rwanda; ^2^Department of Applied Statistics, University of Rwanda, P.O. Box 1514, Kigali, Rwanda; ^3^Department of Veterinary Pathology, Microbiology and Parasitology, University of Nairobi, P.O. Box 29053-00625, Nairobi, Kenya

## Abstract

Canine nematodes pose a public health risk to humans and livestock; however, the prevalence of canine nematodiases in Rwanda is unknown. This study aimed at determining the prevalence of canine nematodiases and identifying the risk factors for such infections in Kigali, the capital city of Rwanda. A cross-sectional study involved 93 dogs selected across Kigali city. Faecal samples were collected from apparently healthy dogs, and nematode eggs were identified and quantified using the McMaster technique. Risk factors for canine nematodiases were analysed by a multivariable binary logistic regression model. The overall prevalence of gastrointestinal (GI) nematodiases in dogs was 33.3% (95% CI: 23.8–42.9). The most prevalent species was *Ancylostoma* spp with 32.3% (95% CI: 22.8–41.8). Nearly 38.7% and 3.2% of the dogs infected with *Ancylostoma* spp and *Toxocara canis* had high egg counts per gram (EPG) of faeces (≥550), respectively. Approximately 96.8% of dogs infected with nematodes had monoinfection. Logistic regression analysis showed that dog's age (1 to 2.5 years old), location (Gasabo and Kicukiro districts), and feeding practices were significantly associated with prevalence of canine nematodiases. In particular, the adjusted odds ratio (AOR) was more than 5 times higher for dogs fed on uncooked animal products and leftovers from households and restaurants compared to those who ate food prepared for them. The AOR was also about 16 times higher for dogs that scavenged and ate leftovers from households compared to those who ate food prepared for them. The findings of this study indicate that the prevalence of GI nematodes in domestic dogs in Kigali city, Rwanda, was 33.3% (95% CI: 23.8–42.9). The identified nematodes, namely, *Ancylostoma* spp. and *Toxocara canis*, are zoonotic, and dogs and humans are at risk of contracting these nematodes. The factors associated with canine GI nematodes in Kigali city include feeding practices and the dog's age and location (district). Dog owners need to rethink procedures for deworming and feeding their dogs. Again, the public should be made aware of the role of dogs in transmitting zoonotic nematodes to humans.

## 1. Introduction

Dogs play a considerable role in helping humans to improve quality of life [1]. It has been demonstrated that pet dog owners are healthier than nonowners [2, 3]. Pet dogs can help people under stressful conditions in enjoying their recreation and curing some pathological conditions such as high blood pressure [4]. Also, people can own dogs for various reasons such as business, hunting, herding livestock, and guarding. Dogs also offer a variety of services such as helping the disabled live independently and search and rescue missions as well as sniffing drugs and explosive detection [5]. Although dogs have become an indispensable companion, they also constitute a potential source of a variety of human infections [1].

Dogs can harbour parasitic infections, thus transmitted between livestock and humans including helminths and protozoa [6, 7]. Ascarids and *Ancylostomatidae* have been frequently reported to be the main helminths of dogs and cats with global significance [8]. Dogs contract ancylostomiasis through skin penetration or oral ingestion of infective larvae. Oral infection occurs through ingestion of infected milk or paratenic hosts or while being suckled [9]. Canine toxocariasis can be transmitted via ingestion of faecal material or soil contaminated with viable embryonated eggs, transplacentally, or ingestion of milk from infected dams while being suckled [10].

Humans can contract nematodes through ingesting items contaminated with embryonated eggs or infective larvae. Also, infective larvae can be transmitted through the cutaneous route as well as mosquito bites (e.g., Filariae). *Ancylostoma* spp that affects dogs can cause cutaneous larva migrans while *Toxocara canis* can cause visceral larva migrans and ocular larva migrans in humans [11, 12]. Studies conducted around the world reported the prevalence of canine GI nematodiases that varied between 9.5% and 51% [13, 14].

A wide range of factors can influence the prevalence of canine helminthiases including intrinsic factors such as age, sex, and breed or extrinsic ones, for instance, feeding, environment, accuracy of testing, regular deworming, and geographical location [15–19]. The control of canine nematodiases and helminthiases at large consists of proper hygiene, regular preventive deworming, and treatment of clinically ill individuals [20, 21]. However, misuse of anthelminthics may lead to the emergence of resistance to the drugs used for the treatment of animal and human helminthiases [20]. Anthelminthic resistance can primarily be prevented through applying evidence-based treatment, respecting the dosage and adherence to proper management strategies [8].

A study conducted in Egypt found that alkaline pH of soil influenced the occurrence of soil-transmitted helminths (e.g., *Toxocara* spp and *Ancylostomatidae*). Soil properties, for instance, temperature, moisture, pH, and organic matter, may influence the prevalence of soil-transmitted helminths [22]. For example, the development of eggs of *Ancylostomatidae* requires a temperature varying between 20 and 30°C and suitable shade and moisture alongside clay-sandy soil [22, 23]. In Rwanda, soils are naturally fragile and derived from schistose, quartzite, gneissic, granite, and volcanic rocks. Again, the country's 88% of the soil pH is acidic (pH 3.5-6.5) [24, 25]. Despite the potential importance of canine nematode infections as a one health concern, there are no reports on their infections in Rwanda. Thus, this study aimed at determining the prevalence of GI nematodes in dogs and associated risk factors in Kigali city, Rwanda.

## 2. Materials and Methods

### 2.1. Study Sites

**Figure 1:**
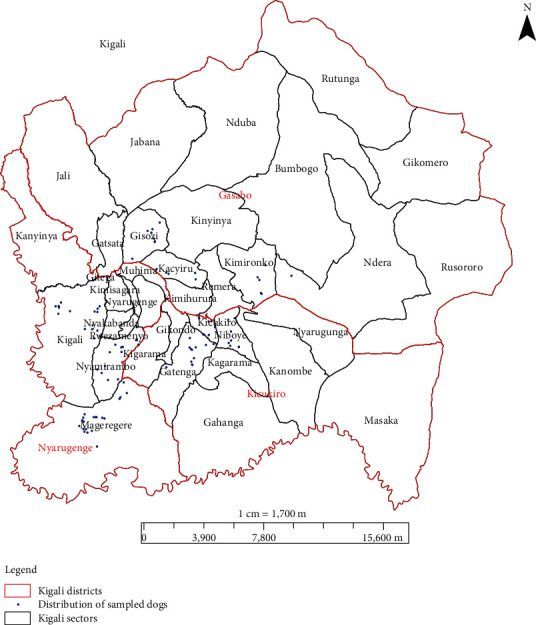
Administrative districts (red boundaries) and sectors (black boundaries) of Kigali city. The blue dots show the location of households owning sampled dogs across the study sites. The locations are Kigali, Nyamirambo, and Mageragere sectors of Nyarugenge district; Kicukiro, Niboye, and Gatenga sectors of Kicukiro district; and Gisozi, Kimironko, and Kacyiru sectors of Gasabo district. Data on the location of each study dog was collected using GPS and allowed generating the map using ArcGis10.2 software.

This study was conducted in Kigali city, Rwanda, from September 2016 to March 2017. In Rwanda, the climate is tropical, and the country has four climatic zones, including the central plateau, where Kigali city is situated [24]. The plateau has an annual rainfall and a temperature ranging from 1,100 to 1,300 mm and 18 to 20°C, respectively [24]. One report showed that the relative humidity in Kigali ranges between 61% and 85% in August and April, respectively [26]. In the suburb of Kigali, soils are hill ferro and valley histosoil types. Much of the soil across Kigali is also acidic [25]. Administratively, Kigali city is subdivided into three districts, and each district is in turn subdivided into administrative sectors [27]. The present study covered nine sectors that were selected from the three districts of Kigali city; each district was represented by three sectors.  Figure 1 shows the map of Kigali city with district and sector level boundaries.

### 2.2. Study Design and Sample Size

This cross-sectional study involved collecting data on management practices and faecal samples from dogs.

We determined the sample size based on the national dog population of 18,117 reported in 2016 [28]. The number of dogs in Kigali represented 2,157, thus 11.9% of the national dog population [29]. Due to a lack of previous studies on canine helminthiases in Rwanda, the prevalence of canine nematodiases was assumed to be 50%.

Based on previous population-based health studies such as the Rwanda demographic and health survey, where the response rate has generally remained above 95% [30], we expected a relatively high response rate. Thus, we increased the sample size by only 10% to cater for possible nonresponse [31, 32]. The minimal sample size (*n*) of dogs needed for testing hypothesis on risk factors for nematodiases in this study was thus estimated using Cochran's formula for determining sample size for proportions [33], as follows:

(1) $$\begin{array}{c} \frac{Z^{2}p\left(1-p\right)/e^{2}}{1+Z^{2}\cdot p\left(1-p\right)/e^{2}N}=\frac{1.96^{2}\times 0.50^{2}/0.10^{2}}{1+1.96^{2}\cdot 0.50^{2}/0.10^{2}\times 2157}=\frac{96.04}{1+96.04/2157}=91.94≅92\text{dogs}, \end{array}$$

where *N* is the population size and *e* is the level of precision. After adjusting for a 10% nonresponse rate (9 additional dogs), the present study targeted a total sample of 101 dogs. Based on district-level registers for the dog population, which showed households owning dogs, we obtained a listing of all dogs for each of the selected sectors. We selected this study sample through a two-stage sampling procedure. In the first stage, we considered the dog population per administrative sector and selected three administrative sectors with the largest dog population from each of the three districts of Kigali city. Thus, we chose nine sectors across Kigali city, namely, Gatenga, Niboye, and Kicukiro in the Kicukiro district; Kacyiru, Kimironko, and Gisozi in Gasabo district; and Mageragere, Nyamirambo, and Kigali in Nyarugenge district. In the second stage, systematic random sampling helped choose the households at the sector level. Cooperation with local authorities allowed locating the target households. Given some households owned many dogs, we randomly considered one dog per household for data collection.

### 2.3. Data Collection

The study was approved by the Rwanda National Ethics Committee (Ethical approval 115/RNEC/2017). Dog owners were explained about this study and signed written consent before participating.

Data were successfully collected from 93 dogs, and a questionnaire was used to collect data on dogs (age, sex, breeds, and location) and on dog keeping practices (frequency of deworming, feeding practices, and control of dog movements) [34]. Faecal samples were collected directly from the rectum using a gloved finger and kept in faecal jars. All samples were stored in a cool box and were analysed at the laboratories of the Rwanda Agriculture and Animal Resources Development Board (RAB).

### 2.4. Faecal Analysis

The preparation of float fluid involved dissolving sodium chloride (Park Scientific Limited, UK) in tap water. The flotation fluid had a specific gravity of 1.200; that is, 400 g of sodium chloride was dissolved in a litre of water. The analysis was done on the day of sampling using the McMaster technique as previously described by Hansen and Perry [35]. Nematode eggs were identified by examining the sample under a light microscope at 10x magnification based on shape, thickness of shell, and presence of morulae [36].

### 2.5. Data Processing and Analysis

Data were entered and then analysed in the IBM SPSS Statistics for Windows, version 20. EPG of faeces was obtained by multiplying the number of eggs by a factor of 50 as previously described by Hansen and Perry [35]. The infection was quantified by EPG which was grouped into light infection (50-100 EPG), moderate infection (150-500 EPG), and heavy infection (≥550 EPG) [37]. The analysis of faecal samples resulted in a binary response variable that indicated whether a sampled dog was infected or not infected. These data were used to determine infection prevalence. To investigate associations between selected factors and prevalence of canine nematodiases, data were analysed using a multivariable binary logistic regression model as described previously [38, 39]. The backward variable selection procedure was applied to selected variables for the best fitting model for the data at hand [40]. The 5% level of significance was used to interpret the results regarding the association. The 95% confidence intervals for the AORs were used to assess the significance and direction of the associations.

## 3. Results

### 3.1. Characteristics of Study Dogs

Faecal samples were collected from 93 apparently healthy dogs from different locations and of different ages, sex, and breeds.

Nearly 15.1% and 29% were <1 year old and 1 to 2.5 years old, respectively, while 31.2% and 24.7% aged >2.5 to 5 years (a dog infected with ascarids belonged to this category) and >5 years, respectively. Some study dogs received regular or irregular deworming. Some dogs were scavengers or fed on food prepared for them or leftovers from households or restaurants. Also, some dogs were restricted while others were not restricted. The occurrence of gastrointestinal nematodes in study dogs is indicated (Table 1).

**Figure 2:**
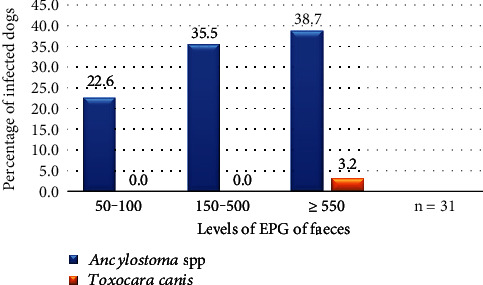
Parasitic egg load in dogs suffering from ancylostomiasis and toxocariasis in Kigali city. Counts ≥ 500 EPG were found in 38.7% of dogs infected with *Ancylostoma* spp. The one dog infected with *T. canis* was also infected with hookworm (1/31) and had count ≥ 500 EPG. In addition, 35.5% and 22.6% of those infected with *Ancylostoma* spp had moderate egg load and light egg load, respectively (Figure 2).

The prevalence of nematodiases in dogs was 33.3% (95% CI: 23.8–42.9). The predominant species was *Ancylostoma* spp with 32.3% (95% CI: 22.8–41.8) (Table 1). Of all the 31 infected dogs, 96.8% were parasitised with one species of nematodes while one dog was infected with two species of nematodes. Parasitic load in dogs suffering from ancylostomiasis and toxocariasis in Kigali city, Rwanda, is shown (Figure 2).

Chi-square tests of the associations of the occurrence of canine nematodiases with the selected potential risk factors in Kigali city and corresponding *p* values are presented in Table 2.

All the variables in Table 2 were considered for the backward variable selection procedure that was based on a multivariable logistic regression model [40]. The results showed that only three factors, namely, feeding practices, dog's age and location, were significantly related to the infection. The results in Table 3 show the AORs and the corresponding 95% confidence intervals.

The results in Table 3 show that the prevalence of canine nematodiases in Kigali city was significantly associated with the dog's age (1 to 2.5 years old), location, and feeding practices. The AOR of nematodiases was more than 10 times higher for dogs that were aged 1-2.5 years (AOR = 10.732; 95% CI: 1.510-76.263) compared to those who were younger than one year old.

Compared to dogs located in Nyarugenge district, the AORs of the infection were about 22 times higher in dogs located in Gasabo district (AOR = 21.617; 95% CI: 4.242-110.160) and about 12 times higher in dogs located in Kicukiro district (AOR = 11.959; 95% CI: 2.534-56.445). Besides, the AOR was more than 5 times higher for dogs fed on uncooked animal products and leftovers from households and restaurants compared to those who ate food prepared for them.

The odds of canine nematodiases were also about 16 times higher for dogs that scavenged and ate leftovers from households compared to those who ate food prepared for them.

## 4. Discussion

The prevalence of canine nematodiases in Kigali city was 33.3% (95% CI: 23.8–42.9). The dog's age, location (district), and feeding practices were statistically significant risk factors associated with the canine nematodiases. Our findings can help policymakers to strategize effective control measures in dog populations and to inform dog owners of the role of dogs in transmitting zoonotic nematodiases to humans.

Our overall prevalence was lower but comparable to 51% reported by Yacob et al. [14] in Ethiopia. However, it was higher than 9.5% reported by Wright et al. [13] in England. Management practices and environmental factors might have influenced the difference in prevalence in these various studies. Around 46.2% of this study dogs received regular or irregular deworming, while 83.9% ate food provided by the owners. However, none of the dogs investigated by Yacob et al. received anthelminthics. Although some of the dogs investigated by Wright et al. got anthelminthics, none received such drugs in the three weeks before faecal sampling. Given *Ancylostoma* spp and *Toxocara canis* are geohelminths, the acidic pH of the soil in Kigali may negatively impact their prevalence. One study found alkaline pH of the soil influences the occurrence of soil-transmitted helminths [22].

The prevalence of *Ancylostoma* spp was comparable to 34.8% found by Davoust et al. [41] in Gabon. However, it was higher than 24.6% reported by Ayinmode et al. [42] in Nigeria and lower than 93.8% reported by Schandevyl et al. [43] in the Democratic Republic of Congo (DRC).

The difference in prevalence may be related to management practices and accuracy in coprological testing. For instance, most dogs involved in the study by Schandevyl et al. [43] in the DRC were not properly looked after and they all were in poor condition. Furthermore, Schandevyl et al. [43] performed both McMaster technique and larval culturing to detect *Ancylostoma* spp.

Different species belonging to *Ancylostomatidae* can infect dogs including *A. caninum*, *A. braziliense*, *A. ceylanicum*, and *Uncinaria stenocephala* [44]. Of these, *A. braziliense*, *A. caninum*, and *U. stenocephala* can cause cutaneous larva migrans (sand-worm disease) in humans, while *A. ceylanicum* can cause eosinophilic enteritis. Similarly, *A. caninum* has been reported to cause eosinophilic enteritis, but it rarely matures into an adult in the human small intestine [12, 45].

Studies conducted in Rwanda reported human ancylostomiasis prevalence that varies between 6.33% and 33% [46, 47]. Counts ≥ 500 EPG were found in 38.7% of dogs infected with *Ancylostoma* spp. One dog infected with *T. canis* was also infected with hookworm and had count ≥ 500 EPG. These dogs could shed a high number of eggs in the environment and potentially put people at risk of contracting ancylostomiasis and toxocariasis.

Given the challenges to distinguish species of *Ancylostoma* based on egg morphometry and that a host can be parasitised by several species concurrently [44], the investigation of human ancylostomiasis in Rwanda should consider the risk of exposure to faeces deposited by walking and wandering dogs. The present study prevalence of *Toxocara canis* was lower than 9.8% reported by Ayinmode et al. [42] in Nigeria. *Toxocara canis* antibodies have been detected in people across Africa. For instance, two previous studies conducted in preschool children aged between 9 months and 5 years old in Nigeria and children aged 1-15 years old in Ghana detected *Toxocara canis* antibodies in 37.3% and 53.5% of the study children, respectively [48, 49]. Further, one study conducted in various groups of professionals in Egypt detected anti-*T. canis* antibodies in 24% of the professionals [50].

Although there are no published data about human toxocariasis in Rwanda, the dog suffering from toxocariasis and ancylostomiasis in this study had an EPG of 750 for *Toxocara canis.* The high level of infection in the tested dog (EPG ≥ 550) suggests that the dog could shed a high number of eggs in the environment and potentially put people at risk of developing visceral larva migrans and ocular larva migrans [12]. Like a previous study by Yacob et al. [14] in Ethiopia, this study found that the dog's age correlated with the prevalence of nematodiases. The present study also found a correlation between the dog's location and the prevalence of canine nematodiases. Besides, our findings were consistent with those of Abere et al. [18] in Ethiopia, who reported that feeding practices positively correlated with the prevalence of canine helminthiases.

Single time point faecal sampling in this study may have impacted the prevalence of canine nematodiases. Thus, the data in this study is a snapshot prevalence which may not necessarily represent the true burden of canine nematodiases. A kinetic study could shed some light on the prevalence and the dynamics of nematodiases in dogs. We also used the egg detection method (McMaster technique), but it would not detect prepatent infections. Even centrifuging faecal samples before examining them can miss numerous hookworm infections [51]. Further studies using molecular laboratory techniques would be more suitable.

## 5. Conclusions

The findings of this study indicate that the prevalence of GI nematodes in domestic dogs in Kigali city, Rwanda, was 33.3% (95% CI: 23.8–42.9). The identified nematodes, namely, *Ancylostoma* spp. and *Toxocara canis*, are zoonotic, and dogs and humans are at risk of contracting these nematodes. The factors associated with canine GI nematodes in Kigali city include feeding practices and the dog's age and location (district). Dog owners need to rethink procedures for deworming and feeding their dogs. Again, the public should be made aware of the role of dogs in transmitting zoonotic nematodes to humans.

## Figures and Tables

**Table 1:** Prevalence of nematodes in analysed faecal samples (*n* = 93 dogs).

Nematode species	Number of infected dogs	Percent (%)	95% CI for percentage	
			Lower limit	Upper limit
*Ancylostoma* spp	30	32.3	22.757	41.759
*Toxocara canis*+*Ancylostoma* spp	1	1.1	0.0	3.171
Total	31	33.3	23.752	42.914

**Table 2:** Chi-square tests of the associations of the occurrence of canine gastrointestinal nematodiases with the selected potential risk factors in Kigali city.

Sample characteristics	Canine nematodes			*p* value
	Present (%)	Absent (%)	Total (%)	
*Deworming frequency*				0.824
Less than twice a year	21 (22.6)	38 (40.9)	59 (63.4)	
At least twice a year	4 (4.3)	9 (9.7)	13 (14.0)	
Irregularly	6 (6.5)	15 (16.1)	21 (22.6)	
*Feeding practices*				0.183
Food prepared for dogs	8 (8.6)	22 (23.7)	30 (32.3)	
Uncooked animal products, leftovers from households and restaurants	15 (16.1)	33 (35.5)	48 (51.6)	
Scavenging and leftovers from households	8 (8.6)	7 (7.5)	15 (16.1)	
*Control of movements*				0.646
Nonrestricted	12 (12.9) 1 (1.1)	21 (22.6) (19.4)	33 (35.5)	
Restricted	19 (20.4)	41 (44.1)	60 (64.5)	
*Breed*				0.873
Local	9 (11.8)	19 (20.4)	28 (30.1)	
Pure or cross	22 (23.7)	43 (46.2)	65 (69.9)	
*Age group*				0.138
<1 year	2 (2.2)	12 (12.9)	14 (15.1)	
1-2.5 years	13 (14)	14 (15.1)	27 (29)	
>2.5-5 years	10 (10.8)	19 (20.4)	29 (31.2)	
>5 years	6 (9.7)	17 (18.3)	23 (24.7)	
*Sex*				0.802
Female	7 (7.5)	17 (18.3)	24 (25.8)	
Male	24 (25.8)	45 (48.4)	69 (74.2)	
*Study district*				0.003
Nyarugenge	8 (8.6)	39 (41.9)	47 (50.5)	
Gasabo	10 (10.8)	9 (9.7)	19 (20.5)	
Kicukiro	14 (15.1)	13 (14)	14 (29)	

**Table 3:** Factors influencing canine nematodiases in Kigali city.

Variable	Categories	Adjusted odds ratio (AOR)	95% C.I. for AOR	
			Lower	Upper
Dog's feeding practices	Food prepared for dogs	1	Reference	
	Uncooked animal products, leftovers from households and restaurants	5.354	1.205	23.776
	Scavenging and leftovers from households	15.646	2.349	104.191
Dog's age	<1 year old	1	Reference	
	1-2.5 years old	10.732	1.510	76.263
	>2.5-5 years old	3.923	0.597	25.779
	>5 years old	4.765	0.643	35.301
Location	Nyarugenge	1	Reference	
	Gasabo	21.617	4.242	110.160
	Kicukiro	11.959	2.534	56.445
	Constant	0.006	—	—

## Data Availability

The data collected and analysed to support the findings of this study are included in the article. The questionnaire used during the data collection is available at https://doi.org/10.21203/rs.3.rs-26155/v4, and the source is cited at a relevant place within the text as a reference [34].
